# Vascular dementia increases levels of methylenetetrahydrofolate reductase and cystathionine β-synthase in female patients and changes gene expression of acetylcholine and glutamate clathrin-sculpted transport vesicles

**DOI:** 10.1101/2025.11.21.689865

**Published:** 2025-11-25

**Authors:** Sanika M. Joshi, Abbey McKee, Sharadyn Ille, Kristina Buss, Thomas G Beach, Geidy E Serrano, Nafisa M. Jadavji

**Affiliations:** 1 College of Osteopathic Medicine, Midwestern University, Glendale, AZ; 2 Department of Biomedical Sciences, College of Dental Medicine, Midwestern University, Glendale, AZ; 3 Bioinformatics Core, Arizona State University, Tempe, AZ; 4 Banner Sun Health Research Institute, Sun City, AZ; 5 Department of Biomedical Sciences, School of Medicine, Southern Illinois University, Carbondale, IL, USA; 6 Department of Neuroscience, Carleton University, Ottawa, Canada; 7 Department of Child Health, College of Medicine – Phoenix, University of Arizona, Phoenix, USA

**Keywords:** One-carbon metabolism, Vascular Dementia, Methylenetetrahydrofolate reductase, Choline, Spatial Transcriptomics

## Abstract

Deficiencies in one-carbon (1C) metabolism are linked to the onset of vascular dementia (VaD). Our previous work using mouse models has demonstrated that reduced dietary intake of folic acid or genetic deficiencies in 1C metabolism result in worse outcomes using a model of VaD. This study aims to provide a detailed molecular portrait of 1C metabolism within the context of VaD, shedding light on potential molecular mechanisms. In cortical post-mortem tissue from female and male VaD patients and controls we measured the folate receptor (FR) and 1C enzymes including methylenetetrahydrofolate reductase (MTHFR), thymidylate synthase (TS), choline acetyltransferase (ChAT), acetylcholinesterase (AChE), cystathionine β-synthase (CBS), with NeuN and DAPI as markers. Additionally, spatial transcriptomics was performed on 4 samples. VaD and gender impacted levels of FR. Both male and female VaD had increased levels of ChAT. Female VaD patients had higher levels of MTHFR and CBS when compared to males. Spatial transcriptomics revealed reduced expression of the glutamate and acetylcholine clathrin-sculpted transport vesicles and increase in glutamatergic neurons. VaD is a complex disease; the results of this study demonstrate that VaD impacts levels of 1C, as well as gene expression. Dietary supplementation with 1C may be beneficial for affected patients.

## 1. Introduction

Vascular dementia (VaD) is projected to double in prevalence within the next three decades and represents a complex and multifaceted clinical syndrome arising from cerebrovascular disease, characterized by cognitive decline and functional impairment [1]. VaD comprises a spectrum of cognitive disorders resulting from cumulative vascular pathologies affecting the brain. The cumulative burden of cerebrovascular lesions, especially intracranial small vessel disease (SVD), correlates with VaD, featuring common occurrences of lacunar infarcts, microinfarcts, and diffuse white matter changes [2].

Several subtypes of VaD have been identified, highlighting the complexity of the relationship between vascular issues and cognitive decline [3]. One notable aspect of VaD is that there is no single neuropsychological pattern that can definitively distinguish it from other causes of cognitive impairment. The underlying vascular pathology of VaD shares pathogenic elements, including hypoperfusion, increased oxidative stress and inflammation, leading to endothelial damage, blood-brain barrier breakdown, innate immunity activation, and disruption of trophic coupling between vascular and brain cells [4]. Nutritional status has been linked to VaD [5].

Deficiencies in one-carbon (1C) metabolism, such as dietary deficiencies in folic acid or choline have been linked to cognitive impairment, including VaD in the elderly population [5,6]. Our work has shown that there is a complex interplay between dietary, genetic, and vascular factors in the manifestation of cognitive deficits [7–9]. We have demonstrated novel insights into arterial remodeling, GFAP expression, and MMP-9 levels, contributing to a more nuanced understanding of the pathophysiological mechanisms underlying VaD [7]. These findings underscore the significance of considering the multifaceted nature of VaD. Another study from our group challenged existing controversies surrounding the association between folic acid metabolism, homocysteine, and VaD, shedding light on the nuanced impact of these factors on cognitive function, vasculature, and choline metabolism [8]. Understanding of the role of 1C metabolism in VaD requires further investigation. The aim of this study was to investigate the levels of 1C enzymes and receptors, as well as gene expression in post-mortem brain tissue from female and male patients diagnosed with VaD.

## 2. Materials and Methods

### 2.1. Brain tissue samples

Human cortical brain tissue was obtained from Banner Sun Health Research Institute Brain and Body Donation Program [10]. These experiments were IRB exempt. Samples were obtained from postmortem female and male patients with a vascular dementia diagnosis and healthy controls. Details of samples including age and comorbidities are listed in Table 1.

### 2.2. Immunofluorescence Experiments

To investigate 1C enzyme levels co-expression within neuronal nuclei, immunofluorescence experiments of ischemic stroke and control brain tissue was performed. Several primary antibodies were used to identify the specific protein in question for a given sample: FR (1:100, Thermo Fisher, Waltham, MA, USA, RRID: AB_2609390), MTHFR (1:100, AbCam, Boston, MA, USA, RRID: AB_2893493), TS (1:100, Cell Signaling, Danvers, MA, USA, catalog # 9045), ChAT (1:100, Millipore Sigma, Darmstadt, Germany, RRID: AB_2079751), AChE (1:100, Millipore Sigma, Darmstadt, Germany, RRID: AB_10602654), CBS (1:100, ThermoFisher, catalog number: MA5–17273) were used. All brain sections were stained with a marker for neuronal nuclei, NeuN (1:200, AbCam, Waltham, MA, USA, RRID: AB_10711040). Primary antibodies were diluted in 0.5% Triton X and incubated on the brain tissue overnight at 4°C. Brain sections were incubated with secondary antibodies Alexa Fluor 488 or 555 (1:200, Cell Signaling Technologies) the following day at room temperature for 2h then stained with 4′, 6-diamidino-2phenylindole (DAPI, 1:10,000).

### 2.3. Immunofluorescence Analysis

Microscope slides were cover slipped with fluromount and stored at 4°C until analysis. The staining was visualized using the Echo Revolve microscope (Echo, San Diego, CA, USA) and all images were collected at the magnification of 200×. To assess 1C protein levels within ischemic stroke and control brain tissue, colocalization of the primary antibody with NeuN and DAPI-labeled neurons was counted and averaged across three sections per primary antibody per subject. Cells were distinguished from debris by identifying a clear cell shape and intact nuclei (indicated by DAPI and NeuN) under the microscope. All cell counts were conducted by two individuals blinded to treatment groups. Using ImageJ, the number of positive cells was counted for a total of 14 healthy aged control subjects (7 males, 7 females) and 16 ischemic stroke subjects (9 males, 7 females).

### 2.4. Statistics for Immunofluorescence Studies

GraphPad Prism 6.0 was used to immunofluorescence staining quantification. In GraphPad Prism 10.6.0, D’Agostino-Pearson normality test was performed prior to two-way ANOVA analysis when comparing the mean measurement of both sex and group (control or VaD). Significant main effects of two-way ANOVAs were followed up with Tukey’s post-hoc test to adjust for multiple comparisons. All data are presented as mean + standard error of the mean (SEM). Statistical tests were performed using a significance level of 0.05.

### 2.5. Spatial Transcriptomics

#### 2.5.1. Sample Preparation

The 10X Genomics Visium Spatial Transcriptome experiment was performed according to the Manufacturer’s inatruction (10X Genomics, Pleasanton, CA, Catalog # 1000520). A total of 4 FFPE cortical brain tissue sections were used for analysis, including female and male VaD and healthy control patients. Briefly, tissue was deparaffinized and stained with H&E and then imaged. Tissue was then de-crosslinked to release mRNA and whole human transcriptome probe panels were hybridized. The Visium CytAssist was used to complete genomic analysis.

#### 2.5.2. Analysis

Spaceranger (from 10X Genomics) was used to integrate the sequencing output with the spatial images to quantify gene expression across each tissue sample. The GRCh38 human transcriptome, formatted for Spaceranger by 10X Genomics, was used as a reference, with the Visium human transcriptome probe set v2.0. Due to difficulties aligning the image for MFG-10–62 (male VaD), the Loupe Browser 7 from 10X Genomics was used to prepare the input for Spaceranger; both cytassist image alignment and manual fiducial alignment were needed for successful alignment.

The R package[11] Seurat [12–15] was used to characterize the expression patterns within each sample with UMAP and tSNE clustering. Differential feature expression was calculated for VaD vs control samples, and the resulting lists of significantly differently expressed genes were submitted to PANTHER [16,17]for a statistical overrepresentation test using Fisher’s Exact test with FDR calculation. Functions with high fold enrichment and low FDR rates were visualized with ggplot2 in R[18].

Using a single-nucleus sequencing database from the Allen Brain Institute (the M1 10X dataset from human brain) as a reference, cell types represented in each spot were deconvoluted and visualized with the R package CARD[19].

## 3. Results

### 3.1. Folate Receptor Levels

**Figure 1.**
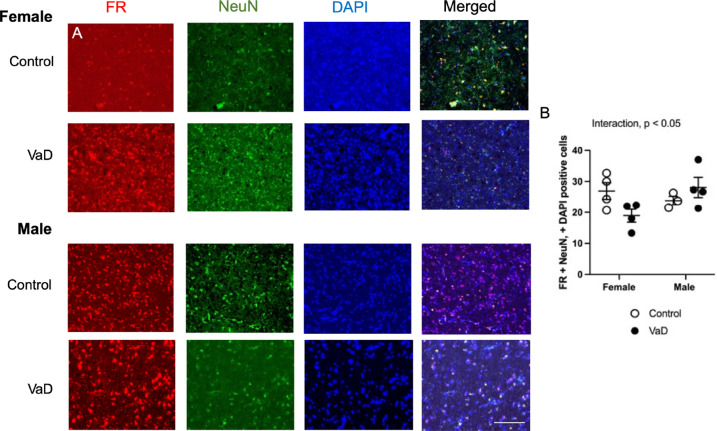
Levels of folate receptor (FR), co-stained with neuronal nuclei (NeuN) and DAPI in brain tissue of controls and Vascular Dementia (VaD) patients. Representative images (A) and quantification of levels (B). Significant interaction between disease and sex, p < 0.05. Data represents 4 patients per group. Scale bar at 50 μm.

Using immunofluorescence, we stained brain tissue from healthy controls and VaD patients. Representative staining from all groups of folate receptor (FR) are shown in Figure 1A. There was a significant interaction between VaD and gender (Figure 1B; F(_1,11_) = 5.41, p = 0.040). There was no difference between genders (p = 0.27) or disease state (p = 0.49).

### 3.2. Thymidylate synthase (TS) levels

TS levels were not impacted by VaD (p = 0.64, data not shown) or gender (p = 0.37). There was no interaction between VaD or gender (p = 0.58).

### 3.3. Methylenetetrahydrofolate reductase (MTHFR) levels

**Figure 2.**
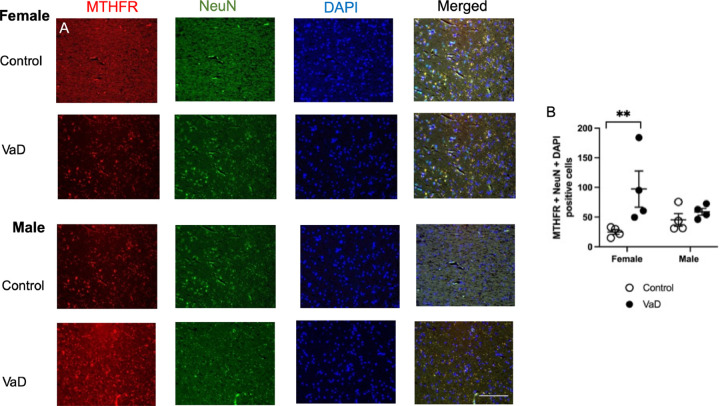
Levels of methylenetetrahydrofolate reductase (MTHFR), co-stained with neuronal nuclei (NeuN) and DAPI in brain tissue of controls and Vascular Dementia (VaD) patients. Representative images (A) and quantification of levels (B). Data represents 4 patients per gender, per group. ** p < 0.01, Tukey’s pairwise comparison between female control and VaD patients. Scale bar at 50 μm.

Representative staining from all groups of methylenetetrahydrofolate reductase (MTHFR) are shown in Figure 2A. MTHFR levels were impacted by VaD (Figure 2B, F(_1,12_) = 5.41, p = 0.023). Female VaD patients had higher levels of MTHFR in cortical tissue compared to healthy controls (p = 0.0092). There was no interaction (p = 0.09) or impact of gender (p = 0.60) on MTHFR levels. We checked for outlier using the GraphPad Quick Cals Grubb’s test. The z was 1.42267 when the p-value was set as 0.05, therefore there was no significant outlier.

### 3.4. Choline acetyltransferase (ChAT) levels

**Figure 3.**
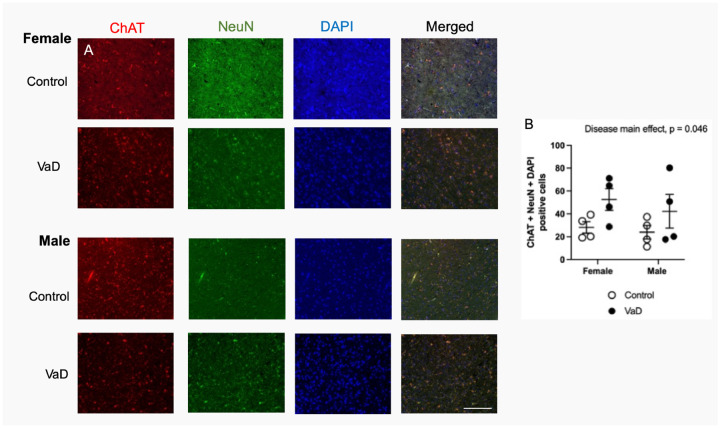
Levels of choline acetyltransferase (ChAT), co-stained with neuronal nuclei (NeuN) and DAPI in brain tissue of controls and Vascular Dementia (VaD) patients. Representative images (A) and quantification of levels (B). Data represents 4 patients per group. Scale bar at 50 μm.

Representative staining from all groups of choline acetyltransferase (ChAT) are shown in Figure 3A. ChAT levels were increased in VaD patients (Figure 3B, F(_1,12_) = 4.95, p = 0.046). There were no differences between groups. There was no interaction (p = 0.75) or impact of gender (p = 0.46) on ChAT levels.

### 3.5. Acetylcholinesterase (AChE) levels

AChE levels were not impacted by VaD (p = 0.45, data not shown) or gender (p = 0.41,). There was no interaction between VaD and gender (p = 0.44).

### 3.6. Cystathionine-β-synthase (CBS) levels

**Figure 4.**
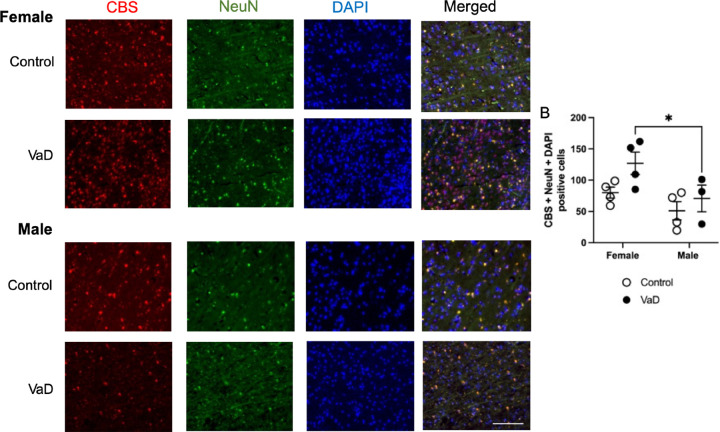
Levels of Cystathionine-ß-synthase (CBS) co-stained with neuronal nuclei (NeuN) and DAPI in brain tissue of controls and Vascular Dementia (VaD) patients. Representative images (A) and quantification of levels (B). Data represents 4 patients per group. * p < 0.05, Tukey’s pairwise comparison between female control and VaD patients. Scale bar at 50 μm.

Representative staining of cystathionine-β-synthase (CBS) is shown in Figure 4A. CBS levels were impacted by gender (Figure 4B, F(_1,11_) = 7.32, p = 0.0204). VaD female had higher levels compared to male patients (p = 0.033). There was no interaction (p = 0.41) or impact of VaD (p = 0.0585) on CBS levels.

### 3.7. Gene Expression

**Figure 5.**
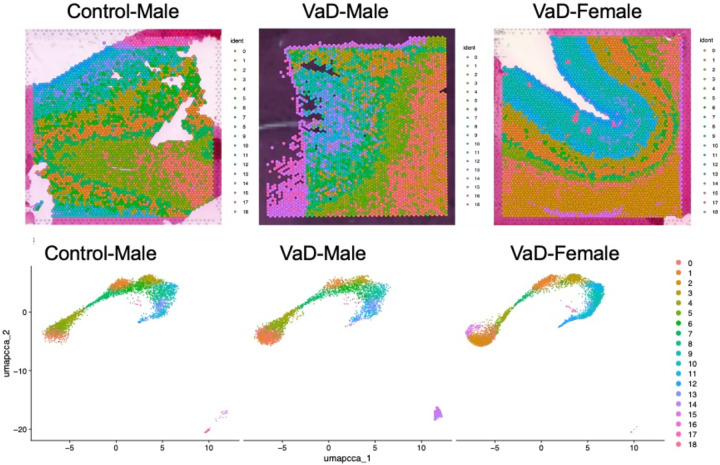
A UMAP (uniform manifold approximation and projection) dimension reduction of clusters across the integrated dataset, showing the conserved clusters between samples. The colors in both the UMAP plots and spatial overlay correspond to the cluster number shown to the right of the image. Each cluster represents a different pattern of gene expression.

Clustering of gene expression patterns shows clear spatial organization within each sample except female healthy control, suggesting potential tissue degradation prior to sequencing or imaging (**supplementary Figure 1**). For this reason, we chose to remove that sample from the analysis, to reduce the risk of incorrect results caused by expression patterns unrelated to dementia. Identified clusters are shown in Figure 5 using the three remaining samples, the cell types were conserved between the tissue samples.

**Figure 6.**
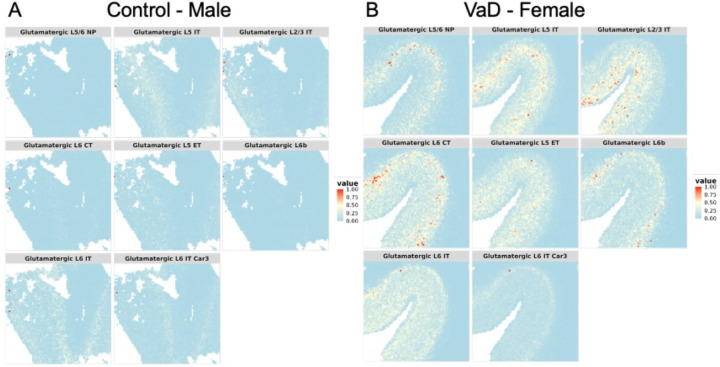
Comparison of cell type proportions across tissue samples for the male control (A) and the female VaD (B) patients. The spatial expression pattern for near-projecting glutamatergic neurons found in the fifth and sixth cortical layers (glutamatergic L5/6 NP) and intratelencephalic-projecting glutamatergic neurons found in the fifth cortical layer (glutamatergic L5 IT) provide an alignment between the two tissues, since they are both present to a higher degree in both tissues. Most of the other types of glutamatergic neurons have increased presence in the VaD patient.

Deconvolution of cell types by cluster was performed and shows which cell types correspond to the different clusters, and therefore to the different spatial layers within each tissue. Comparing cell-type expression across the spatial images allows alignment and reorientation of each tissue slice compared to the others. Looking at the patterns of oligodendrocyte and astrocyte presence in the sample, the left edge and lower right corner of the healthy control male slice are seen to be equivalent to the inner curve of VaD female. Using that reference as an anchor, the visualization then shows increased levels of multiple cell types in the female VaD patient. While demyelination caused by the loss of oligodendrocytes is associated with VaD [20], the cell type proportions in these tissues don’t show a clear difference between the control and female VaD patient. However, the increased presence of multiple types of glutamatergic neurons in the VaD patients (Figure 6).

### 3.8 Differential Functional Analysis

**Figure 7.**
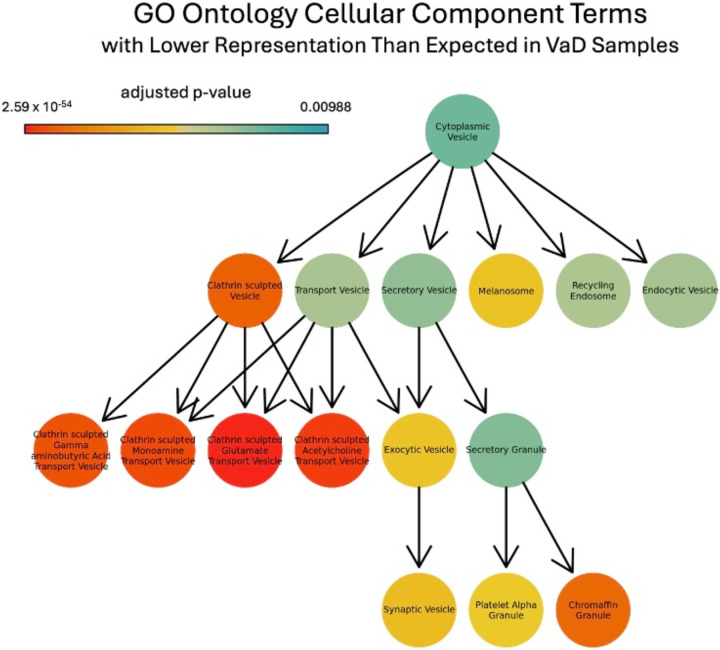
Portion of the cellular component gene ontology (GO)[21] hierarchy tree showing functional terms that were significantly under-represented in VaD samples, colored by adjusted p-value.

Comparison of control and VaD patients showed decreased representation of multiple functional terms in the VaD samples (Figure 7). There is lower-than-expected representation of clathrin-sculpted transport vesicles, including those for glutamate and acetylcholine. We did not observe any changes in expression levels of MTHFR, ChAT, and CBS between VaD and healthy controls. The expression was also not clustered spatially across tissue.

Functional overrepresentation in control compared to VaD patients’ analysis further shows decreased representations of functions related to axonal transport and sodium-potassium transporter activity (Supplemental Table 1). There was over-representation of genes annotated with negative regulation of amyloid beta formation as well as its precursor processes, implying a connection between VaD and amyloid beta, this connection has been discussed at length previously [4] (Supplemental Table 2).

## 4. Discussion

To further elucidate the intricate relationship between one-carbon (1C) metabolism and vascular dementia (VaD), the present study aimed to investigate the levels of 1C enzymes and the folate receptor in post-mortem cortical brain tissue from VaD and control patients. We also measured spatial transcriptomics in brain tissue of VaD and healthy control patients. Our analysis shows that the folate receptor levels were impacted by VaD and gender. Female VaD patients had increased levels of MTHFR and CBS. VaD impacted choline metabolism was impacted only by levels of ChAT which makes acetylcholine at synapses. Our spatial transcriptomics analysis demonstrated interesting changes in glutamine as well as cellular components. There were reduced levels of acetylcholine in VaD patients, which corresponds to increased levels of ChAT in cortical brain tissue. Our previous work [8,22], as well as others [23]has shown reduced levels of acetylcholine result in increased levels of ChAT.

Homocysteine is marker of 1C metabolism as well as disease state [9]. Clinical studies have revealed a relative risk of dementia in the elderly with moderately raised homocysteine within the normal range, ranging from 1.15 to 2.5, and a Population Attributable risk from 4.3% to 31%[5]. Intervention trials with B vitamins in elderly individuals with cognitive impairment demonstrate a significant slowing of whole and regional brain atrophy, along with a deceleration of cognitive decline [24]. These findings suggest that moderately raised plasma total homocysteine (>11 mol/L), prevalent in the elderly, may contribute to age-related cognitive decline and dementia. The International Consensus Statement, drawn from a comprehensive review of literature spanning the last two decades, emphasizes the importance of identifying modifiable risk factors for the prevention of dementia [5]. The public health significance of addressing elevated homocysteine is underscored, given its ease, cost-effectiveness, and safety of treatment with B vitamins. In 2024, the Food for the Brain foundation launched a home test to measure homocysteine levels in the elderly, with the goal to catch impaired 1C metabolism and correct it with dietary supplementation [25]. Further trials are recommended to explore whether B vitamin treatment can slow or prevent the conversion to dementia in individuals at risk of cognitive decline or dementia.

VaD comprises a spectrum of cognitive disorders resulting from cumulative vascular pathologies affecting the brain. It can be challenging to identify the clinical and pathological substrates of cerebrovascular disease in the context of VaD, with vascular changes often found in the elderly either alone or alongside neurodegenerative processes [2]. Additionally, several subtypes, highlighting the complexity of the relationship between vascular issues and cognitive decline [3]. Our work has added to the literature and confirmed previous findings. Our spatial transcriptomics work specifically confirmed the previous finding that clathrin itself is significantly downregulated in VaD [26]. While demyelination caused by the loss of oligodendrocytes is associated with VaD [27], our data did not don’t show a clear difference between the control and VaD patient. This could be a result of the tissue we analyzed. Additionally, there was an increased presence of multiple types of glutamatergic neurons in the VaD patient is consistent with past studies that have shown altered glutamate metabolism in the VaD population [28].

Choline is an essential nutrient found in food that is a precursor in the synthesis of phospholipids, betaine, and acetylcholine. Betaine is a methyl group donor for homocysteine to methionine reaction. Acetylcholine is an important neurotransmitter involved in neurogenesis, synapse formation, learning and memory [29]. Changes in choline metabolism have been reported in VaD [30–32]. Our work confirms these changes both through immunofluorescence experiments and spatial transcriptomics. Choline is made endogenously in our body, but we require dietary supplementation – and the neuroprotective effects of increasing choline have been shown by our group using a mouse model of ischemic stroke [33,34], as well as others [30]. Recent reports show that ~90% of the US population does not reach the acceptable daily intake (ADI) of choline [35].

To follow up clinical findings that increased levels of homocysteine were linked to cognitive impairment [36], several research groups have shown that changes in 1C impact cognitive impairment [7–9,37,38]. In our opinion investigating the link of 1C and VaD in post-mortem brain tissue was the next step. Our present study shows that the FR and MTHFR, ChAT, and CBS enzymes are impacted by VaD. Interestingly there is also a sex difference, which has also been shown in the clinical population [39]. We measured levels of enzymes in cortical tissue, investigating other areas of the brain as well as markers in blood, such as homocysteine would also provide more data.

## 5. Conclusions

VaD is a complex disease; the results of this study demonstrate the impact of VaD on 1C and changes in gene expression. Supplementation with 1C in VaD patients may be beneficial for VaD affected patients as well as targeting reduced gene expression to increase better outcomes.

## Supplementary Material

**Supplementary Materials:** Supplementary Figure 1, Supplementary Tables 1 and 2

## Figures and Tables

**Table 1.** Patient sample demographics and comorbidities.

	Control		VaD	
Age	Male	Female	Male	Female
>90+	1/8 (13%)	3/8 (38%)	0/8 (0%)	3/8 (38%)
<90	3/8 (38%)	1/8 (13%)	4/8 (50%)	1/8 (13%)
**Comorbidities**				
CAA	2/8 (25%)	2/8 (25%)	0/8 (0%)	1/8 (13%)
HR	-	-	0/8 (0%)	1/8 (13%)
LBS	-	-	2/8 (25%)	1/8 (13%)

Abbreviations: CAA - Cerebral amyloid angiopathy; HR - Hippocampal Sclerosis, LBS- Lewy Bodies; VaD – vascular dementia

## Data Availability

Corresponding author can be contacted for data.

## Highlights

### What are the main findings?

1. In post-mortem cortical tissue, we report increased levels of enzymes and the folate receptor and choline acetyltransferase in female and male patients with Vascular Dementia. Female VaD patients had increased levels of methylenetetrahydrofolate reductase (MTHFR) and cystathionine β-synthase (CBS).
2. Using spatial transcriptomics, we show reduced levels of glutamate and acetylcholine clathrin-sculpted transport vesicles and increase in glutamatergic neurons in VaD patients.

### What is the implication of the main finding?

1. Vascular dementia leads to an increase in the demand of one-carbon metabolism.
2. One-carbon metabolism has a wide breath of impact on cellular function. Monitoring levels of homocysteine, a marker of one-carbon metabolism status, maybe an effective clinical tool to reduce negative implications.

## Abbreviations

The following abbreviations are used in this manuscript:

AChE: Acetylcholine Esterase
CAA: Cerebral amyloid angiopathy
CBS: Cystathionine beta synthetase
ChAT: Choline acetyltransferase
FR: Folate receptor
HR: Hippocampal Sclerosis
LBS: Lewy Bodies
MTHFR: methylenetetrahydrofolate reductase
NeuN: Neuronal nuclei
TS: Thymidylate synthase
VaD: vascular dementia
